# 3D facial landmarks: Inter-operator variability of manual annotation

**DOI:** 10.1186/1471-2342-14-35

**Published:** 2014-10-11

**Authors:** Jens Fagertun, Stine Harder, Anders Rosengren, Christian Moeller, Thomas Werge, Rasmus R Paulsen, Thomas F Hansen

**Affiliations:** 1 Department of Applied Mathematics and Computer Science Technical University of Denmark, Richard Petersens Plads, Building 324, DK-2800, Lyngby, Denmark; 2 Institute for Biological Psychiatry Mental Health Center Sct Hans Copenhagen University Hospital, Boseupvej 2, DK-4000, Roskilde, Denmark

**Keywords:** 3D Facial landmarks, Inter-operator annotation variance, Dense point correspondence, Point distribution mode, ANOVA

## Abstract

**Background:**

Manual annotation of landmarks is a known source of variance, which exist in all fields of medical imaging, influencing the accuracy and interpretation of the results. However, the variability of human facial landmarks is only sparsely addressed in the current literature as opposed to e.g. the research fields of orthodontics and cephalometrics. We present a full facial 3D annotation procedure and a sparse set of manually annotated landmarks, in effort to reduce operator time and minimize the variance.

**Method:**

Facial scans from 36 voluntary unrelated blood donors from the Danish Blood Donor Study was randomly chosen. Six operators twice manually annotated 73 anatomical and pseudo-landmarks, using a three-step scheme producing a dense point correspondence map. We analyzed both the intra- and inter-operator variability, using mixed-model ANOVA. We then compared four sparse sets of landmarks in order to construct a dense correspondence map of the 3D scans with a minimum point variance.

**Results:**

The anatomical landmarks of the eye were associated with the lowest variance, particularly the center of the pupils. Whereas points of the jaw and eyebrows have the highest variation. We see marginal variability in regards to intra-operator and portraits. Using a sparse set of landmarks (n=14), that capture the whole face, the dense point mean variance was reduced from 1.92 to 0.54 mm.

**Conclusion:**

The inter-operator variability was primarily associated with particular landmarks, where more leniently landmarks had the highest variability. The variables embedded in the portray and the reliability of a trained operator did only have marginal influence on the variability. Further, using 14 of the annotated landmarks we were able to reduced the variability and create a dense correspondences mesh to capture all facial features.

## Background

The research field of facial morphology has advanced rapidly over the last ten years, with the introduction of better, faster, and cheaper systems for facial 3D scanning. The systems have enabled more accurate and objective methods of capturing differences in facial morphology. Analysis of facial morphology is based on facial distances i.e. the distance between facial landmarks [1-3] or on statistical models [1,4]. One widely used statistical method, uses Principal Component Analysis (PCA) to assess the population variance and is referred to as a Point Distribution Model (PDM) [5]. Both methods rely on manually annotated landmarks that are used directly or as a basis for constructing a dense point correspondence [1,4-6]. This means that both direct distances and statistically based methods are prone to human operator annotation errors. There exist several surface-based automatic registration methods for point correspondence, still for manual annotation, at least on a sparse set of landmarks, is widely used when facial analysis is used in clinical applications. Understanding the variance (noise) introduced by manually annotated landmarks is important for knowing the statistical power of such studies, i.e. the interpretation and application, and aiding future study design in this field.

The reliability of facial landmark annotation has not been as thoroughly studied as landmark annotations in other fields, e.g. cephalometry [7]. For example, Buschang *et al.*[8] assessed the inter-operator annotation variability of anatomical landmarks on the skull for use in orthodontics and cephalometric analysis, using ANOVA analysis. Similarly, recent have also addressed the reliability of cranial-anatomical landmarks [9-11]. By Larsen et al. the inter-operator annotation variance was included in the PCA when analyzing cranial growth [12]. Here the landmark variance was addressed using a weighting scheme giving most weight to annotation landmarks with low variance.

In this study, we exclusively work with human facial features. We address the reliability of facial feature annotation with respect to inter/intra operators and samples (portraits). To the best of our knowledge, this is the first report on variability of face morphology with respect to the measurements of the face surface, *per se*. In effort to reduce annotation variability i.e. reduce the signal to noise ratio, we suggest a sub-set of landmarks that yields a superior dense-point correspondence compared to the original landmarks, based on the reliability of facial landmarks.

## Methods

### Sample and image data

The data used in this work consists of 36 facial scans of healthy unrelated subjects, recruited among volunteers in the Danish Blood Donor Study (DBDS) [13]. The 36 subjects were chosen by simple random sampling from our database consisting of facial scans from 641 subjects, having 50% males. The facial scans were captured using a Canfield Vectra M3 Imaging System, at the DBDS facility at Glostrup University Hospital. Each 3D facial scan contains about 70,000 to 100,000 3D points and has shape information (x-, y-, z-point positions) and texture information (red, green, blue intensities) for every 3D point. The study was approved ethically by the Danish Scientific Committee and was reported to the Danish Data Protection Agency. All the patients have given written informed consent prior to inclusion in the project. The facial image used in figures, is a statistically average face and does picture any participant.

### Description of annotation points

**Figure 1:**
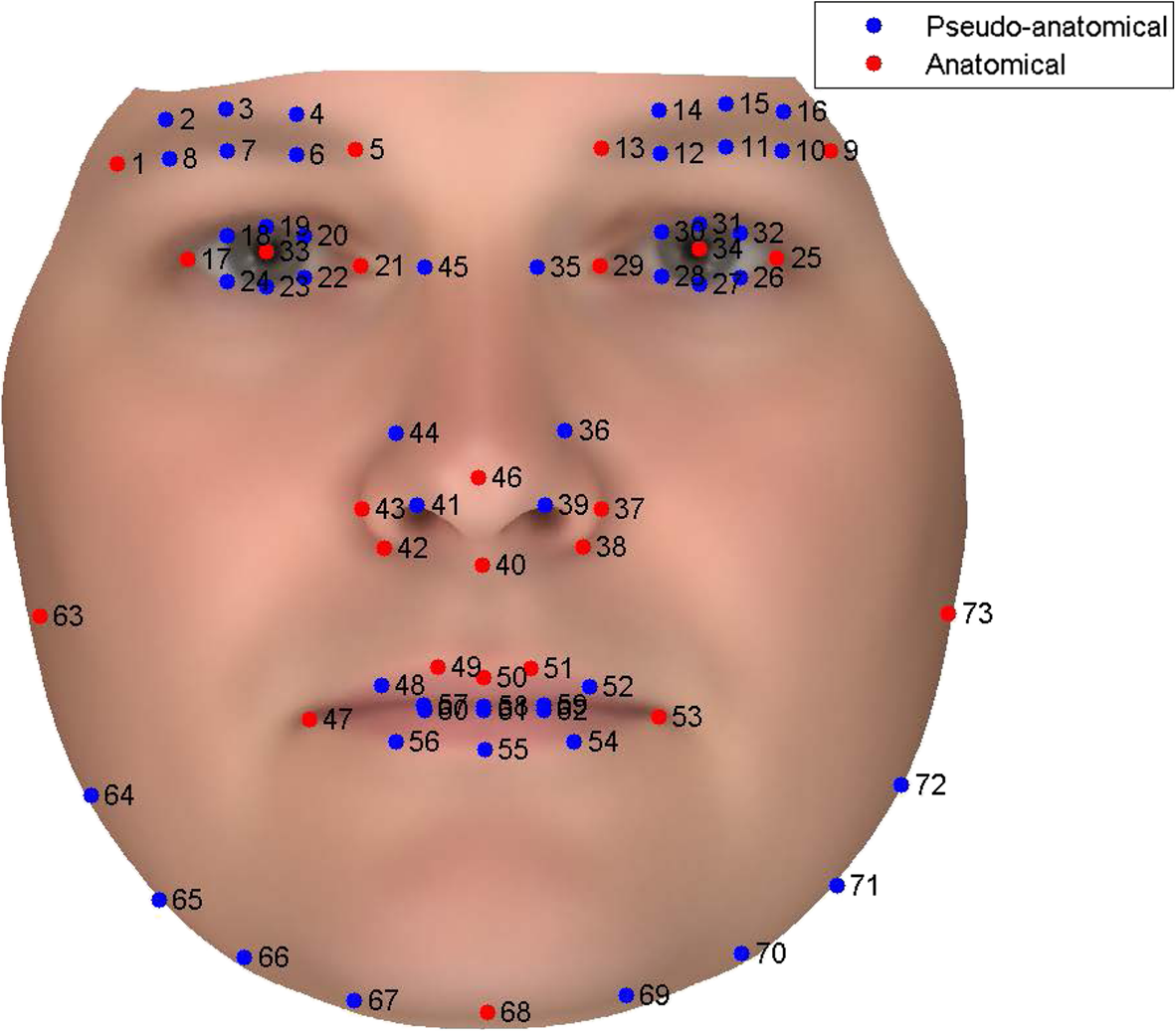
**Facial landmarks divided into anatomical and pseudo-anatomical landmarks.** Anatomical landmarks: 1) Right eyebrow lateral point, 5) Right eyebrow medial point, 9) Left eyebrow lateral point, 13) Left eyebrow medial point, 17) Right eye lateral canthus, 21) Right eye medial canthus, 25) Left eye lateral canthus, 29) Left eye medial canthus, 33) Right pupil center, 34) Left pupil center, 37) The outer left alar-facial groove, 38) The inner left alar-labial groove, 40) The columellars connection to the upper lip, 42) The inner right alar-labial groove, 43) The outer right alar-facial groove, 46) Tip of the nose, 47) Right oral commissure, 49) The right philtrum column connection to the the vermillion border, 50) Midpoint on the cupid’s bow, 51) The left philtrum column connection to the the vermillion border, 53) Left oral commissure, 63) Right ear attachment, 68) Lowest point of the central jaw, 73) Left ear attachment.Pseudo-landmarks groups: 2-4 & 6-8) Right eyebrow, 10-12 & 14-16) Left eyebrow, 18-20 & 22-24) Right eye, 26-28 & 30-32) Left eye, 35 & 36 & 39 & 41 & 44 & 45) Nose, 48 & 52 & 54-62) Mouth, 64-67 & 69-72) Jaw.

The annotation framework initially developed by Fagertun et al. consists of 73 landmarks [14]. Here, 24 anatomical landmarks define distinct facial features, and 49 pseudo-landmarks define the curves and width of the jaw, lips, eyebrows etc. A description is presented in Figure 1.

### Annotation procedure

All scans followed a three-step annotation scheme: 

1. Automated annotation of landmarks (see section “Data pre-processing by automatic annotations”): 

• A fully automatic Active Appearance Model (AAM) in 2D [15].

• An Active Shape Model (ASM) in 3D [16].

2. Correction by human operator of the pre-annotated landmarks (see section “Manual annotation tools and standard”)

3. Post processing (see Section “Dense point correspondence”) 

• Creation of dense point correspondence meshes.

### Data pre-processing by automatic annotations

A 2D image was created by orthographic projection of the 3D scan. The face and eyes are automatically detected by a Viola-Jones Rapid Object Detection [17,18], and serve as a starting point for an AAM search. When the AAM converges, the 73 2D annotation points (Figure 1) can be extracted. These annotation points are then transformed from the 2D image to the 3D scan. The 2D to 3D transformation is likely to fail in high curvature areas like the jaw as points from 2D images are wrongly projected onto the neck. To compensate for this limitation, an ASM search, initialized by an Iterative Closest Point search [19], is performed to locate the jaw in 3D. The annotation points are then manually corrected by an operator see section “Manual annotation tools and standard”. In summary, the low curvature points are found by a 2D AAM and transformed to 3D image, while high curvature points are found by a 3D ASM.

The 2D AAM and 3D ASM were constructed based on 605 individuals recorded by a Nikon D90 in 2D and a Canfield Vectra M3 Imaging System in 3D, respectively. Both the 2D and 3D data were annotated to create correspondence between individuals, in the same fashion as described in the following section.

### Manual annotation tools and standard

The object of the manual annotation was to reach a consistent and stable standard for annotation. Prior to the study the annotation scheme was explained and discussed during a three-hour workshop (common training program), to ensure a common frame of reference. Further, all operators had annotated more than 100 scans prior to this training program. The manual annotation is a two-step process. First, the annotation is performed in a fixed frontal view by a custom-made annotation tool. The fixed view was chosen over free flying mode to allow faster annotation time. Second, points in high curvature areas are adjusted in fixed frontal, profile, and top/down views. The high curvature points are the jaw and nose points (35-45 and 63-73).

### Dense point correspondence

To analyze facial shape variation at positions not annotated by landmarks, a dense point correspondence is created. A variety of methods exist for establishing dense correspondence. In this work we employ a method that has previously produced excellent results when a sparse set of landmarks exist [6].

This method is based on propagating a well-formed template mesh to all shapes in the training set. For each shape the template mesh is initially deformed using a volumetric thin-plate spline warp [20] and using the sparse set of corresponding landmarks. In the next step the mesh vertices of the deformed template mesh are propagated to the target shape. This approach is very similar to the method used to create the dense surface models described by Hutton *et al.*[1,4,5]. While propagating each vertex to the Euclidian closest point on the target surface works for simple anatomy, it fails in regions with moderate curvature. A proven solution is to regularize the correspondence field and add curvature information in the propagation step. In Paulsen [6] and Hilger [21] this regularization is cast into a Markov Random Field (MRF) framework [22], where a prior and an observation term are defined. The prior model imposes a Gaussian prior on the deformation field that favors smooth deformation fields. The curvature of the deformed template mesh and the target shape is used in the observation term to guide the correspondence to areas with similar curvature. The mean curvature is estimated as the radius of a locally fitted sphere [23]. Finally, the regularization is bounded so the projected points are on the surface of the target shape. The optimal correspondence field is found using stochastic optimization. The involved weighting between the prior and observation terms is found as the weight that creates the most compact shape model as described by Hilger [21]. The result is a regularized dense correspondence between the template and all the shapes in the training set. In our experiments, the dense correspondence consists of 39,653 points and the associated mesh connectivity from the template mesh.

### Software

All results were produced with SAS version 9.4 and Matlab version R2010b.

## Results

### Landmark variability

**Figure 2:**
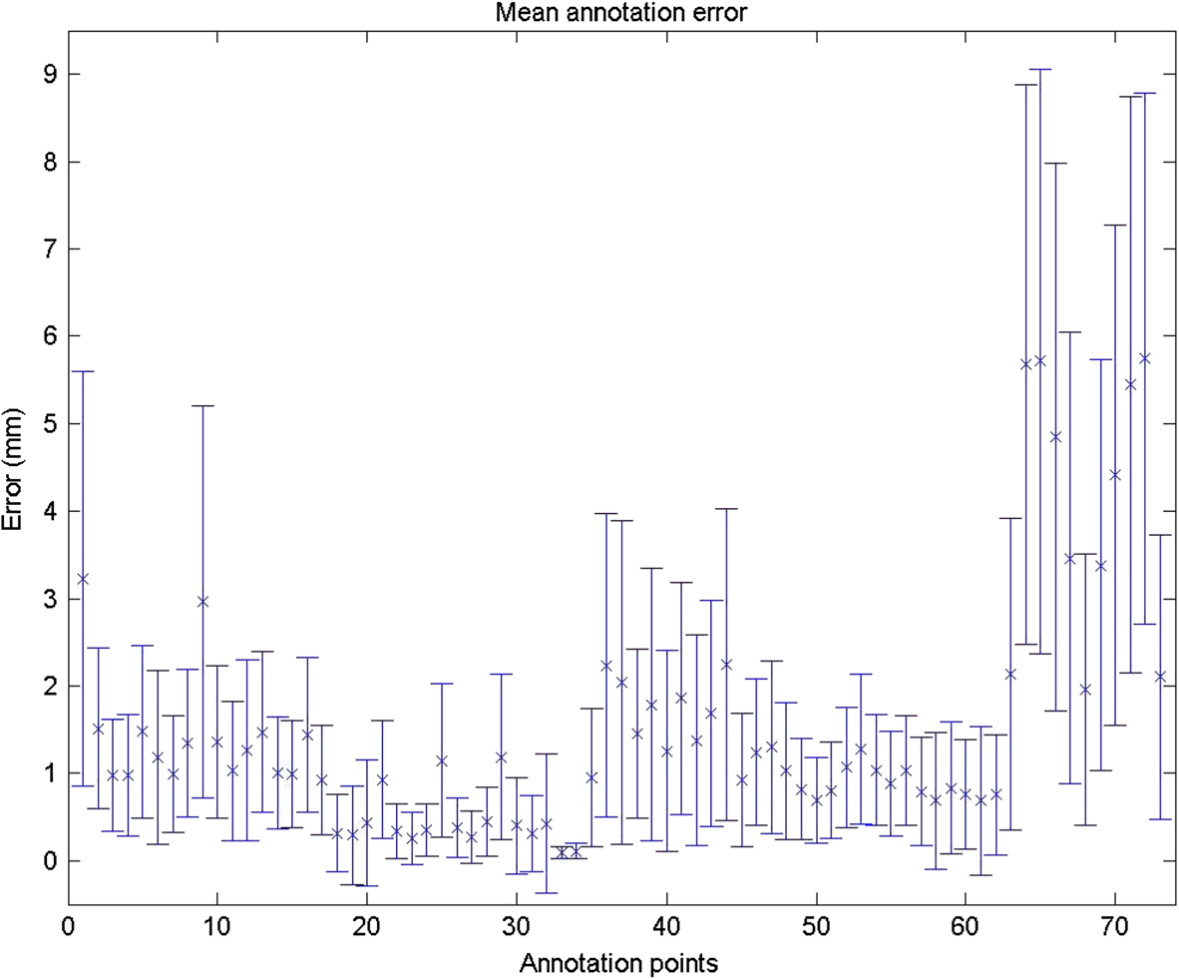
Mean annotation error.

**Figure 3:**
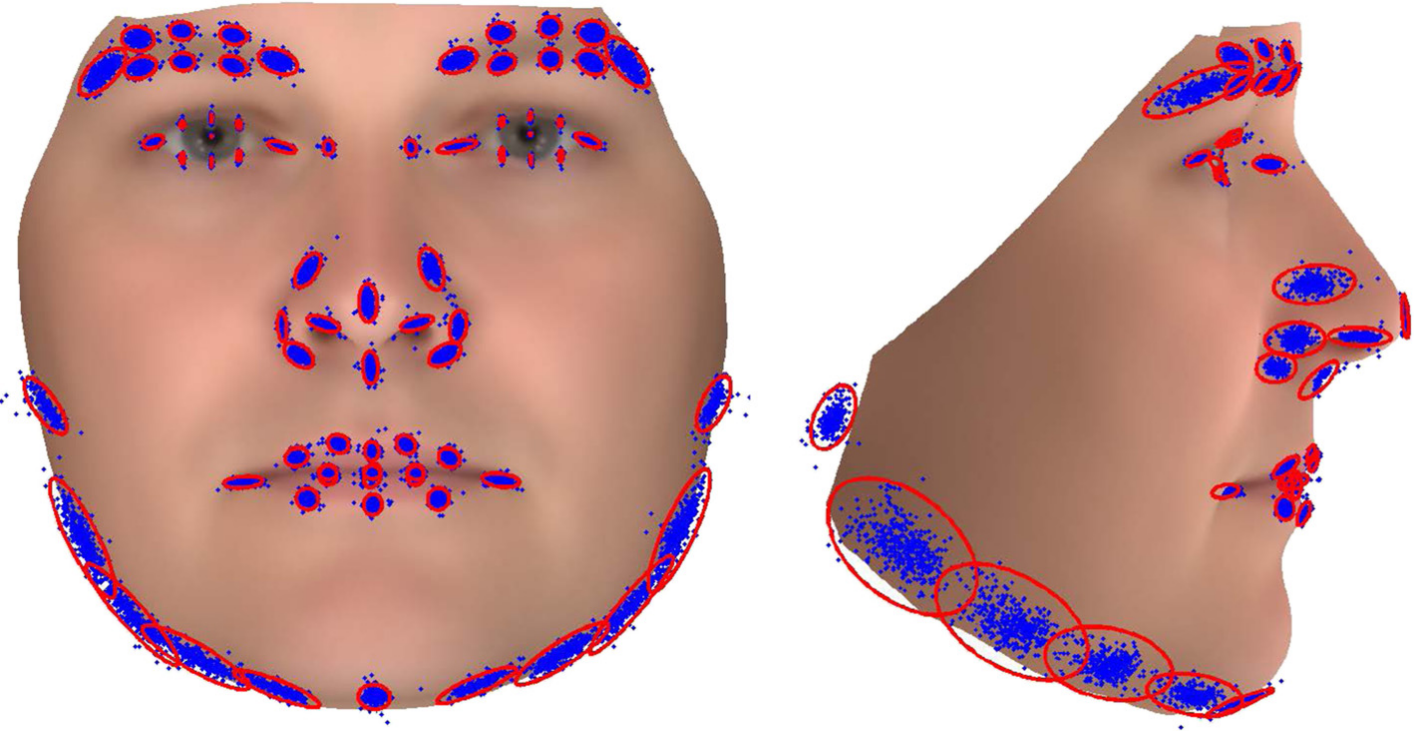
**Frontal and profile annotation variance plot.** Red ellipse shows 3 standard deviations.

Six operators(one female) annotated 36 scans (50% male, aged 18 to 65) twice, one week apart. All six operators went through a common training program and were unblinded to the study aim. The mean error and standard deviation of the combined variance of each annotation point are shown in Figure 2. We observed a association between the variability and the the specific annotation point. The center of the pupil was associated with minimal variance (SD = 0.09 mm), followed by landmarks of the eye (SD = 0.30-0.95 mm). The most error-prone annotation points are the landmarks of the jaw (SD = 1.55-3.34 mm), although the lateral points of the eyebrows are also error prone (SD = 2.24-2.37 mm). The variance of each annotation point is illustrated in Figure 3.

#### Intra/inter operator variability

**Figure 4:**
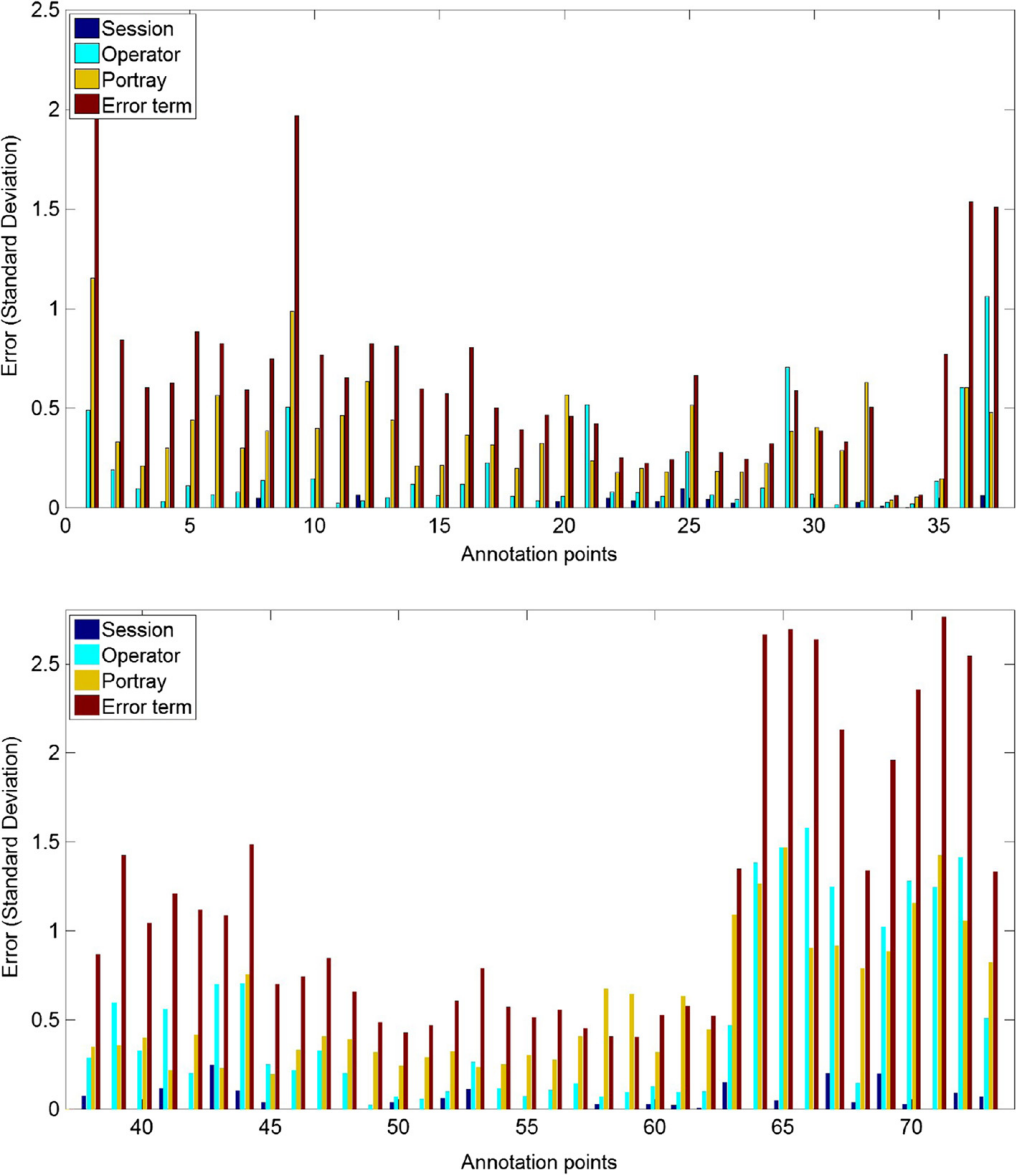
The variance of the operator, session day and portrait.

We used a mixed-model ANOVA analysis, using the Minimum Variance Quadratic Unbiased Estimation (MIVQUE) method to estimate the effects of the components: operator, session day, and the scan number (portrait), for each of the 73 annotated points:

(1) $$Y_{\text{ijk}}=\mu +O_{i}+D_{j}+I_{k}+\varepsilon_{\text{ijk}}$$

where *Y*_*ijk*_ is the data sample, *μ* is the global average, *O*_*i*_, *D*_*j*_ and *I*_*k*_ are the main effect terms for inter-operator, intra-operator (session day) and portrait (individual capturing age and gender), respectively. *ε*_*ijk*_ is the error term for unexplained variance. Three-way ANOVA using interaction terms was rejected as the model did not contribute with further explanation of the variance, data not shown.Generally the session day (i.e. intra-operator) contributed relatively little to the variability, see Figure 4. The most reliable annotation landmark was the center of the pupil, as this was only marginally influenced by the inter/intra-operator and portrait, and was not associated with a large error term. While no significant difference in variance was observed between landmarks and pseudo-landmarks, the variance was more prominent in the points describing the jaw and nose and to some extent the medial canthus of the eye, Figure 4.

### Statistical model fit

In order to test the PDM stability for different operator annotations we adapted a coupled leave-one-out cross-validation scheme. We built a PDM for a single operator and a PDM from a random sampling (using the same number of scans as the former) for the remaining five operators. The PDM’s are built on 35 individuals and the reconstruction error is measured on the 36th individual. We then loop over all individuals in the inner leave-one-out cross-validation loop and over all operators in the outer leave-one-out cross-validation loop. The mean reconstruction error to the mean annotation points for all six operators is presented in Table 1. The table shows that the PDM constructed by random operators is consistently better at reconstructing the annotations. Interestingly no single operator yields a PDM that perform better than the randomly selected PDM.

**Table 1:** Mean annotation reconstruction errors in mm

**Operator**	**1**	**2**	**3**	**4**	**5**	**6**	**Mean**
Single operator	1.466	1.577	1.503	1.502	1.465	1.530	1.507
Random sampling	1.431	1.435	1.421	1.465	1.451	1.393	1.433

The mean annotation errors are shown for a PDM build using a single operator and for a PDM build from a random sampling of the remaining five operators.

### Dense point correspondence optimization

**Figure 5:**
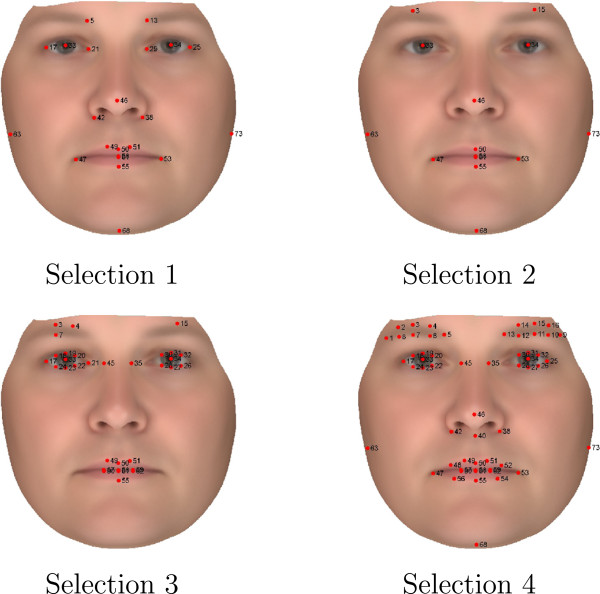
**Landmark selections 1,2,3, and 4.** Selections 1 and 2: Selected for having low mean error and variation with focus on capturing central and all facial features, respectively. Selection 3: Mean error below 1 mm. Selection 4: Operator standard deviation below 0.5 mm.

The 73 annotated points were associated with different variability. We tested four different sub selections of these annotation points in a effort to minimize the variance of the resulting dense point correspondence. Two landmark selections simply excluded annotation points with the highest variance in mean error (>1 mm) and operator error (>0.5 mm), respectively. Two landmark selections which aim at selecting landmarks from the main facial features and with low mean error and variance are also tested, see Figure 5.

The quality of the derived dense point correspondence was evaluated by the size of variance between the correspondence points. If the points had good correspondence, the resulting variation of the correspondence points will manly describe the difference in the samples (population variance). In the case of poor correspondence, the variation will now account for both the population variance and the inconsistency of correspondence points leading to a higher variation. We measured the dense mean point variance for each annotation point and the four suggested landmark selections (Tables 2 and 3).

**Table 2:** Dense point mean variance

**Landmark selections**	**Full**	**1**	**2**	**3**	**4**
Mean variance	1.92	0.62	**0.54**	1.03	0.71

Bold number indicates the lowest mean variance.

**Table 3:** Selection 2’ prediction error and inter-operator error of all 73 landmarks

**Annotation point**	**In seletion**	**Operator error**	**Scheme 2 error**
1	4	3.23	4.87
2	4	1.51	1.69
3	2;3;4	0.98	0.98
4	3;4	0.98	1.70
5	1;4	1.48	2.22
6	4	1.19	1.77
7	3;4	0.99	1.67
8	4	1.35	2.20
9	4	2.96	6.71
10	4	1.36	2.23
11	4	1.03	1.57
12	4	1.26	1.97
13	1;4	1.47	2.31
14	4	1.00	1.55
15	2;3;4	0.99	1.03
16	4	1.44	1.95
17	1;3;4	0.93	2.16
18	3;4	0.31	1.31
19	3;4	0.29	1.48
20	3;4	0.44	1.04
21	1;3	0.93	1.85
22	3;4	0.34	0.81
23	3;4	0.26	0.91
24	3;4	0.35	1.12
25	1;4	1.15	2.48
26	3;4	0.38	0.92
27	3;4	0.27	1.05
28	3;4	0.45	0.82
29	1	1.19	1.07
30	3;4	0.40	1.07
31	3;4	0.31	1.11
32	3;4	0.42	1.40
33	1;2;3;4	0.09	0.17
34	1;2;3;4	0.11	0.39
35	3;4	0.95	1.46
36		2.24	2.05
37	1	2.04	2.23
38	4	1.45	2.35
39		1.79	1.40
40	4	1.26	1.48
41		1.86	1.41
42	1;4	1.38	1.84
43		1.68	2.10
44		2.25	1.91
45	3;4	0.92	1.77
46	1;2;4	1.24	1.26
47	1;2;4	1.30	1.30
48	4	1.03	1.79
49	1;3;4	0.82	0.96
50	1;2;3;4	0.69	0.71
51	1;3;4	0.80	1.26
52	4	1.07	1.53
53	1;2;4	1.28	1.26
54	4	1.04	1.95
55	1;2;3;4	0.88	0.91
56	4	1.03	1.69
57	3;4	0.79	0.94
58	1;2;3;4	0.69	0.69
59	3;4	0.83	1.04
60	3;4	0.76	1.07
61	1;2;3;4	0.69	0.79
62	3;4	0.75	0.82
63	1;2;4	2.14	4.77
64		5.68	4.04
65		5.72	4.13
66		4.85	3.75
67		3.46	3.03
68	1;2;4	1.96	1.97
69		3.38	2.95
70		4.41	3.33
71		5.45	4.92
72		5.75	4.57
73	1;2;4	2.11	7.09

**Figure 6:**
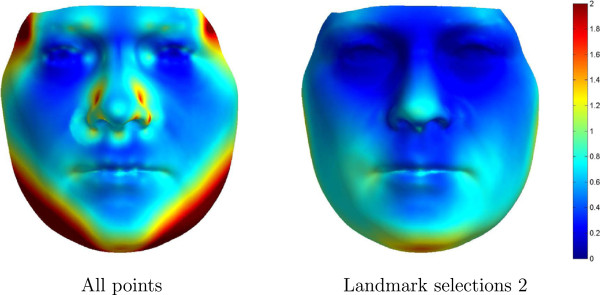
**Visualization of variance.** Variance is displayed in mm of one standard deviation.

The lowest variance is seen for landmark selection 2, having a mean variance = 0.54 mm. Figure 6 illustrates the variation of the PDM from landmark selection 2 compared to the original PDM (based on all landmarks). Annotated points with relatively small inter-operator variation, was not estimated better automatically. However, points with large inter-operator variation was better estimated. Based on these results we conclude that a reduced set of the original 73 landmarks provides optimal annotation. It is also noted that landmark selection 2, which consists of 14 landmarks, results in a more compact model and improves estimation of 16 of the 73 landmarks compared to the manual operator annotations, see (Table 3).

## Discussion

To the best of our knowledge, this is first study to address the variation of human-annotated 3D facial landmarks. Understanding the variation of manual annotations is important as components of registration, recognition, and machine learning are influenced by manual annotation errors. However, the current literature is sparse in area pertaining to 3D facial morphology and variation. We expect that an increase in the availability, accuracy, user friendliness (i.e. fewer operator demands) of 3D imaging scanners will probe the use of shape models in clinical diagnostics, as seen for example in orthopedic surgery [24]. However, to assess the putative clinical impact of such tools, it is important to understand the variability embedded in manual annotation. Our analysis focused on facial morphology, suggests a procedure to retrieve a dense correspondence mesh of the face with low variance and minimal human operator assigned annotation points.

We first address the variability of 73 facial 3D landmarks, and that the variability is highly correlated with specific annotation point. As expected, landmarks that are easier to define in consensus (here, landmark of the pupils) have the lowest inter- and intra-operator variability. More leniently defined landmarks such as the points defining the jaw line are associated with the highest variation. The portray itself was associated with relative low annotations variability, thus is seems that variables associated with the portray such as age and gender does not seem to influence the annotations.

One obvious application of the annotated points is to identify minor facial abnormalities,that may assist in the clinical diagnosis of syndromes. Such abnormalities can be identified by using absolute measures or the ratio between manually annotated landmarks, or by using a dense correspondence mesh. Our study supports the preferential use of dense correspondence mesh for identification of minor abnormalities, as this facilitates the use of landmarks/points not manually annotated and thus a larger data set. In a clinical setting, different operators will be used, and although such operators will be ideally trained, the variability will lead to increased signal to noise ratio and reduced analytical power. Therefore, we suggest an approach to limit the number of annotation points, which minimize variability and is able capture facial features. This approach uses 14 landmarks to create a dense correspondence mesh with a point mean variance of 0.54. Further, this approach shows less variability in 16 of the manually annotated points not included creating the correspondence mesh. Using fewer annotation points will decrease the operator time, thus improving feasibility of use.

There is one obvious limitation with regard to generalizability of the study. We used subjects that are Caucasian with Scandinavian background, thus we cannot exclude that the variability of the annotation landmarks is different from other ethnicities, e.g. the texture of blonde eyebrows on light skin may be difficult to separate, whereas dark eyebrows may not. One other limitation of our study is that annotation was performed only two times, thus we cannot address whether additional repeat measure (>2) would notable influence the annotation variation.

## Conclusion

We found that the variability of manual annotated facial landmarks, was associated with the specific landmark, and did not seem to be influence by the portray, i.e. gender and age, or the (trained) operator. Using 14 of the 73 landmarks we were able to decreasing the mean variance and create a dense correspondence mesh capturing all facial feature.

## Competing interests

The authors declare that they have no competing interests.

## Authors’ contributions

JF data analysis/interpretation and drafted the manuscript. JF, RRP and TFH study design, data interpretation, edited manuscript. SH, AR,CM and TW performed the data acquisition and help with data interpretation. All authors are responsible for the content of the paper and approved the final draft.

## Pre-publication history

The pre-publication history for this paper can be accessed here:

http://www.biomedcentral.com/1471-2342/14/35/prepub
